# iNOS Ablation Does Not Improve Specific Force of the Extensor Digitorum Longus Muscle in Dystrophin-Deficient mdx4cv Mice

**DOI:** 10.1371/journal.pone.0021618

**Published:** 2011-06-30

**Authors:** Dejia Li, Jin-Hong Shin, Dongsheng Duan

**Affiliations:** Department of Molecular Microbiology and Immunology, University of Missouri, Columbia, Missouri, United States of America; Istituto Dermopatico dell'Immacolata, Italy

## Abstract

Nitrosative stress compromises force generation in Duchenne muscular dystrophy (DMD). Both inducible nitric oxide synthase (iNOS) and delocalized neuronal NOS (nNOS) have been implicated. We recently demonstrated that genetic elimination of nNOS significantly enhanced specific muscle forces of the extensor digitorum longus (EDL) muscle of dystrophin-null mdx4cv mice (Li D et al *J. Path.* 223:88–98, 2011). To determine the contribution of iNOS, we generated iNOS deficient mdx4cv mice. Genetic elimination of iNOS did not alter muscle histopathology. Further, the EDL muscle of iNOS/dystrophin DKO mice yielded specific twitch and tetanic forces similar to those of mdx4cv mice. Additional studies suggest iNOS ablation did not augment nNOS expression neither did it result in appreciable change of nitrosative stress markers in muscle. Our results suggest that iNOS may play a minor role in mediating nitrosative stress-associated force reduction in DMD.

## Introduction

Duchenne muscular dystrophy (DMD) is an X-linked lethal muscle disease affecting approximately 1–3 of every 10,000 newborn boys [1]. The primary genetic defect of DMD is dystrophin gene mutation [2]. Dystrophin is a sub-sarcolemmal structural protein essential for muscle cell membrane integrity and signal transduction. In the absence of dystrophin, muscle cells undergo degeneration and necrosis and eventually are replaced by fibrotic and fatty tissues. It is currently not completely clear how the lack of dystrophin leads to this devastating cascade of events. Several mechanisms have been proposed including contraction-induced sarcolemmal rupture, pathogenic calcium overloading, free radical injury, ischemia, inflammation and aberrant signaling (reviewed in [3], [4], [5]).

Recent studies suggest that inducible nitric oxide synthase (iNOS) may represent a common link among several of these proposed mechanisms [6]. iNOS is a calcium-insensitive NOS [7], [8]. Its expression is negligible under normal condition but iNOS is highly up-regulated in inflamed tissues. In dystrophin-deficient mdx mice and DMD patients, iNOS level is markedly elevated in muscle [6], [9], [10], [11]. It is currently not completely clear whether iNOS elevation merely represents an inflammatory signature of muscular dystrophy or it directly contributes to muscle disease in DMD. A recent study by Bellinger et al suggests that iNOS may play an active role in DMD pathogenesis [6].

In normal muscle, the ryanodine receptor (RyR) regulates calcium release from the sarcoplasmic reticulum (SR). When RyR is S-nitrosylated, it becomes leaky. Excessive entry of SR calcium into the cytosol activates calcium-dependent calpain proteases and causes muscle damage and force reduction [12]. Bellinger et al observed a disease-associated RyR S-nitrosylation in the extensor digitorum longus (EDL) muscle of mdx mice. Interestingly, they also found a simultaneous increase of iNOS expression and formation of an iNOS-RyR complex. Based on these findings, the authors proposed that iNOS-mediated RyR S-nitrosylation and subsequent intracellular calcium leaking represent important downstream events in dystrophin-deficient muscular dystrophy. Strategies to reduce iNOS-mediated RyR hypernitrosylation and/or RyR calcium channel leaking may ameliorate DMD [6]. In support of this model, Bellinger et al indeed found that pharmacological inhibition of RyR leaking improved voluntary exercise and EDL muscle specific force in mdx mice [6].

In accordance with these findings, here we hypothesize that genetic elimination of iNOS may improve EDL muscle contractility in dystrophin-null mice, presumably via reduced RyR S-nitrosylation. To test this hypothesis, we crossed the C57Bl/6 (BL6) background iNOS knockout (KO) mice with the BL6 background mdx4cv mice. Progeny mice were genotyped by PCR. After confirming dystrophin and iNOS expression by western blot, we examined the histopathology and contractile profile of the EDL muscle in age-matched male BL6, mdx4cv, iNOS KO and iNOS/dystrophin double knockout (iNOS/Dys DKO) mice. Much to our surprise, ablating iNOS did not reduce histological signs of muscle damage neither did it alter specific muscle forces. BL6 and iNOS KO yielded similar specific twitch and tetanic forces. In mdx4cv and iNOS/Dys DKO mice, specific forces were significantly lower than those of normal. However, there was no significant difference between mdx4cv and iNOS/Dys DKO mice. Interestingly, iNOS/Dys DKO mice appeared slightly more resistant to eccentric contraction-induced injury. To further probe this intriguing finding, we examined nNOS expression and muscle nitrosative stress markers. We did not find evidence of nNOS up-regulation in iNOS-null normal and dystrophic mice. Nitro-tyrosine, total cellular ryanodine receptor 1 (RyR1) and S-nitrosylated RyR1 levels were not altered by iNOS ablation either. Our results suggest that iNOS may be less important than it has been suggested in modulating force generation in dystrophin-deficient muscle.

## Materials and Methods

### Animals

**Figure 1:**
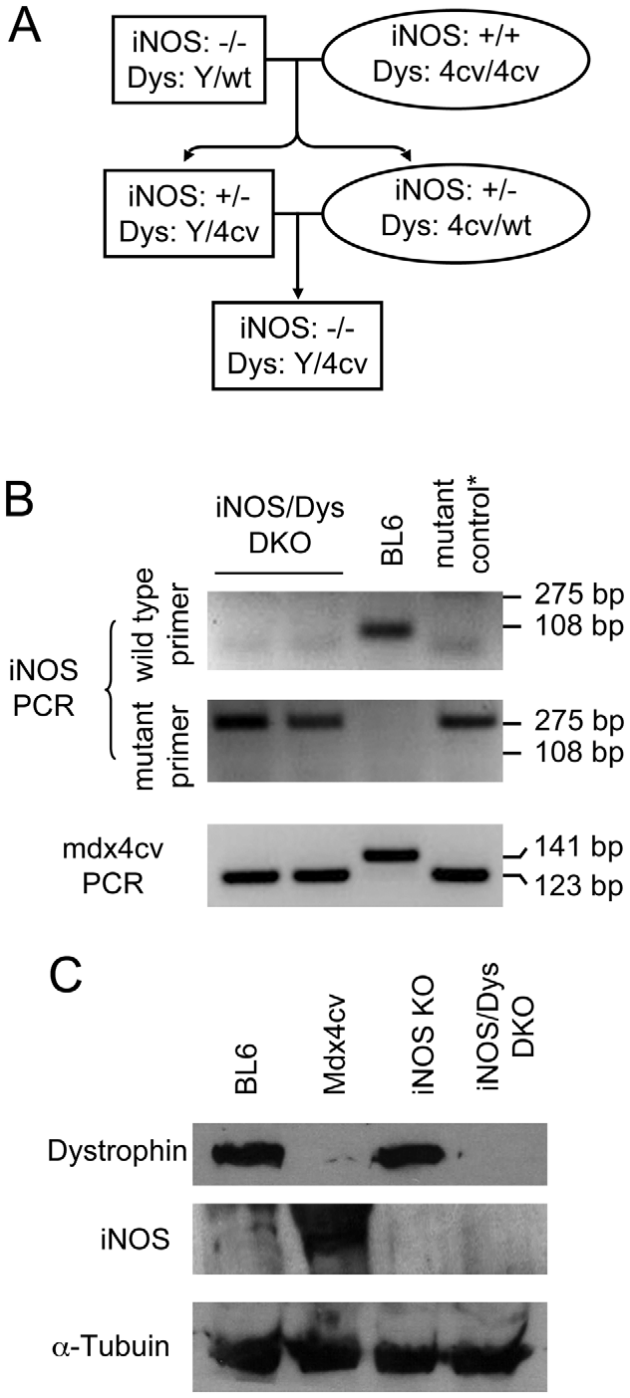
Generation of iNOS/dystrophin double knockout mice. **A**, Outline of the breeding scheme. Dystrophin and iNOS heterozygous male (Dys, Y/4cv; iNOS, +/−) and female (Dys, wt/4cv; iNOS, +/−) mice were generated by crossing iNOS knockout mice with mdx4cv mice. Crossing among heterozygous mice resulted in iNOS/dystrophin double deficient male mice. Rectangle, male mice; Oval, female mice. **B**, Representative genotyping photomicrographs. Top panel, iNOS PCR. Wild type yields a 108 bp band. Knockout yields a 275 bp band. Bottom panel, mdx4cv PCR. Wild type yields a 141 bp band. Mdx4cv yields a 123 bp band. In iNOS PCR, the mutant control is an iNOS knockout mouse. In mdx4cv PCR, the mutant control is a mdx4cv mouse. The first two lanes show typical results from two independent iNOS/dystrophin double knockout mice. **C**, Representative western blot results. DKO, double knockout; Dys, dystrophin; KO, knockout; wt, wild type.

All animal experiments were approved by the Animal Care and Use Committee of the University of Missouri (#6980) and were in accordance with NIH guidelines. BL6, B6.129P2-Nos2tm1Lau/J (iNOS KO) and (B6Ros.Cg-*Dmdmdx-4Cv*/J (mdx4cv) mice were purchased from The Jackson Laboratory (Bar Harbor, ME). Experimental iNOS/Dys DKO mice were generated by crossing iNOS KO and mdx4cv mice (Figure 1A). The genotype of the iNOS locus was determined using a protocol provided by The Jackson Laboratory (http://jaxmice.jax.org/strain/002609.html). Briefly, two independent PCR reactions were conducted using a common primer (ACATGCAGAATGAGTACCGG) and a wild type allele specific primer (TCAACATCTCCTGGTGGAAC) or a mutant allele specific primer (AATATGCGAAGTGGACCTCG). The wild type allele yielded a 108 bp band and the mutant allele yielded a 275 bp band (Figure 1B). The mdx4cv genotype was determined by primer competition PCR as we recently reported [13]. The primers include a common primer (GCGCGGCTTGCCTCTGACCTGTCCTAT), a wild type allele specific primer (GATACGCTGCTTTAATGCCTTTAAGAACAGCTGCAGAACAGGAGAC) and an mdx4cv allele specific primer (CGGCCAGAACAGCTGCAGAACAGGAGAT). The wild type allele yielded a 141 bp band and the mdx4cv allele yielded a 123 bp band (Figure 1B). The average age of the experimental mice was 9.0±0.7 months (range, 6 to 12 months). Only male mice were used in the study.

### Western blot

Whole muscle lysate was obtained from frozen limb muscles [14]. Dystrophin was detected with a monoclonal antibody against the dystrophin C-terminal domain (Dys2, 1∶100, clone Dy8/6C5, IgG1; Novocastra, Newcastle, UK). iNOS was detected with a rabbit polyclonal antibody (#482728, 1∶1,000, EMD Chemicals, Gibbstown, NJ). nNOS was detected with a rabbit polyclonal antibody against the N-terminal end of nNOS (1∶1,000; Upstate, Lake Placid, NY) [14], [15], [16]. Nitro-tyrosine was detected with a mouse monoclonal antibody (1∶1,000; Caymen Chemicals, Ann Arbor, MI) [14]. RyR1 was detected with a mouse monoclonal antibody (1∶1000; Affinity Bioreagents, Golden, CO). For total cellular RyR1, the sarcoplasmic reticulum membrane fraction was prepared as described by Saito et al in the presences of 1% protease inhibitor (Roche, Indianapolis, IN) [14], [17]. For S-nitrosylated RyR1, the sarcoplasmic reticulum membrane fraction was further purified using a resin-assisted capture method as reported by Forrester et al [14], [18].

Rapid blue staining of duplicated gels (Geno Technology, St Louis, MO) was used as loading control for S-nitrosylated RyR1. For all other western blots, membrane was probed with an anti-α-tubulin antibody as the loading control (1∶3,000; clone B-5-1-2; Sigma, St Louis, MO).

### Histology, immunostaining and nNOS activity staining

Morphological studies were performed in the EDL and tibialis anterior (TA) muscles. Both muscles mainly consist of fast-twitch type II myofibers. Haematoxylin and eosin (HE) staining was used to reveal general histology and centrally nucleated myofibers. Sarcolemmal integrity was assessed with the IgG infiltration assay [14], [19], [20]. Briefly, an Alex594 conjugated rabbit anti-mouse IgG antibody (1∶100; Invitrogen-Molecular Probe, Carlsbad, CA) was applied to 8 µm muscle cross sections. After washing, damaged myofibers were visualized as red color under the Texas red channel with a Nikon E800 fluorescence microscope. Macrophage infiltration was determined by immunohistochemical staining using the Vectastain ABC kit (Vector Laboratories, Burlingame, CA) [21], [22]. The murine-specific anti-macrophage antibody (1∶500; rat anti-mouse F4/80) was obtained from Caltag Laboratories (Burlingame, CA). Macrophage stained in dark brown color. Fibrosis was examined with Masson trichrome staining according to our published protocol [14], [21], [23], [24]. Fibrous tissue stained in blue color. Enzymatic nNOS activity staining was performed as previously described [14], [15], [16].

### In vitro evaluation of the EDL muscle force

Twitch and tetanic (50, 80, 120, and 150 Hz) forces of the EDL muscle was measured in vitro at 30°C using a 300B dual-mode servomotor transducer (Aurora Scientific, Inc., Aurora, Ontario, Canada). The force data was analyzed using a DMC/DMA software (Aurora Scientific) [25], [26], [27], [28]. Muscle cross-sectional area (CSA) was calculated according to the following equation, CSA = (muscle mass)/(0.44×Lo×muscle density). 0.44 represents the ratio of muscle fiber length to optimal length for the EDL muscle. Muscle density is 1.06 g/cm^3^. The specific force (kN/m^2^) was calculated by normalizing the absolute muscle force with the CSA. After tetanic force measurement, the muscle was rested for 10 min and then subjected to eccentric contraction injury according to our previously published protocol [25], [26], [27], [28]. The percentage of force drop following each round of eccentric contraction was recorded.

### Statistical analysis

Data are presented as mean ± standard error of mean. Statistical analysis was performed with the SPSS software (SPSS, Chicago, IL). Statistical significance was determined by one-way ANOVA followed by Bonferroni post hoc analysis. Difference was considered significant when *P*<0.05.

## Results

### Generation of iNOS/Dys DKO mice

To eliminate potential influence of the genetic background, we crossed iNOS KO with mdx4cv mice (Figure 1A). Both strains were on the BL6 background. PCR genotyping revealed the loss of wild type iNOS allele and the presence of iNOS KO allele and mdx4cv mutation in iNOS/Dys DKO mice (Figure 1B) [13]. To further confirm the absence of iNOS and dystrophin in iNOS/Dys DKO mice, we performed western blot (Figure 1C). Dystrophin was detected in BL6 and iNOS KO, but not mdx4cv and iNOS/Dys DKO muscle lysates. BL6 muscle showed nominal iNOS expression [29]. As expected, iNOS level was substantially elevated in mdx4cv muscle but was completely eliminated in iNOS KO and iNOS/Dys DKO muscle (Figure 1C) [10].

### Body weight and the anatomic properties of the EDL muscle

Adult male mice (9.0±0.7 months) were used in the study. No significant difference was observed in body weight among BL6, mdx4cv, iNOS KO and iNOS/Dys DKO mice (Table 1). The EDL muscle optimal length did not show significant difference either (Table 1). The EDL muscle weight and cross-sectional area (CSA) were significantly increased in mdx4cv mice (Table 1) [14], [28], [30]. The EDL muscle of iNOS KO mice had similar weight and CSA to those of BL6 mice [31], [32]. Genetic elimination of iNOS significantly reduced the EDL weight and CSA in mdx4cv mice. However, they were still significantly higher than those of normal mice (Table 1).

**Table 1:** Body weight and EDL muscle characterization.

Strain	N	Body Weight (g)	Weight (mg)		Lo (mm)	CSA (mm^2^)	
BL6	11	39.23±1.97	11.14±0.33		13.06±0.08	1.83±0.05	
Mdx4cv	11	37.18±1.41	18.50±0.28	a	13.79±0.14	2.88±0.04	a
iNOS KO	5	32.93±0.56	12.58±0.41		13.18±0.11	2.05±0.06	
iNOS/Dys DKO	4	35.85±0.35	15.85±0.39	a	13.71±0.07	2.48±0.05	a

a Significantly different from all other strains.

### Characterization of muscle histopathology in iNOS/dystrophin double deficient mice

**Figure 2:**
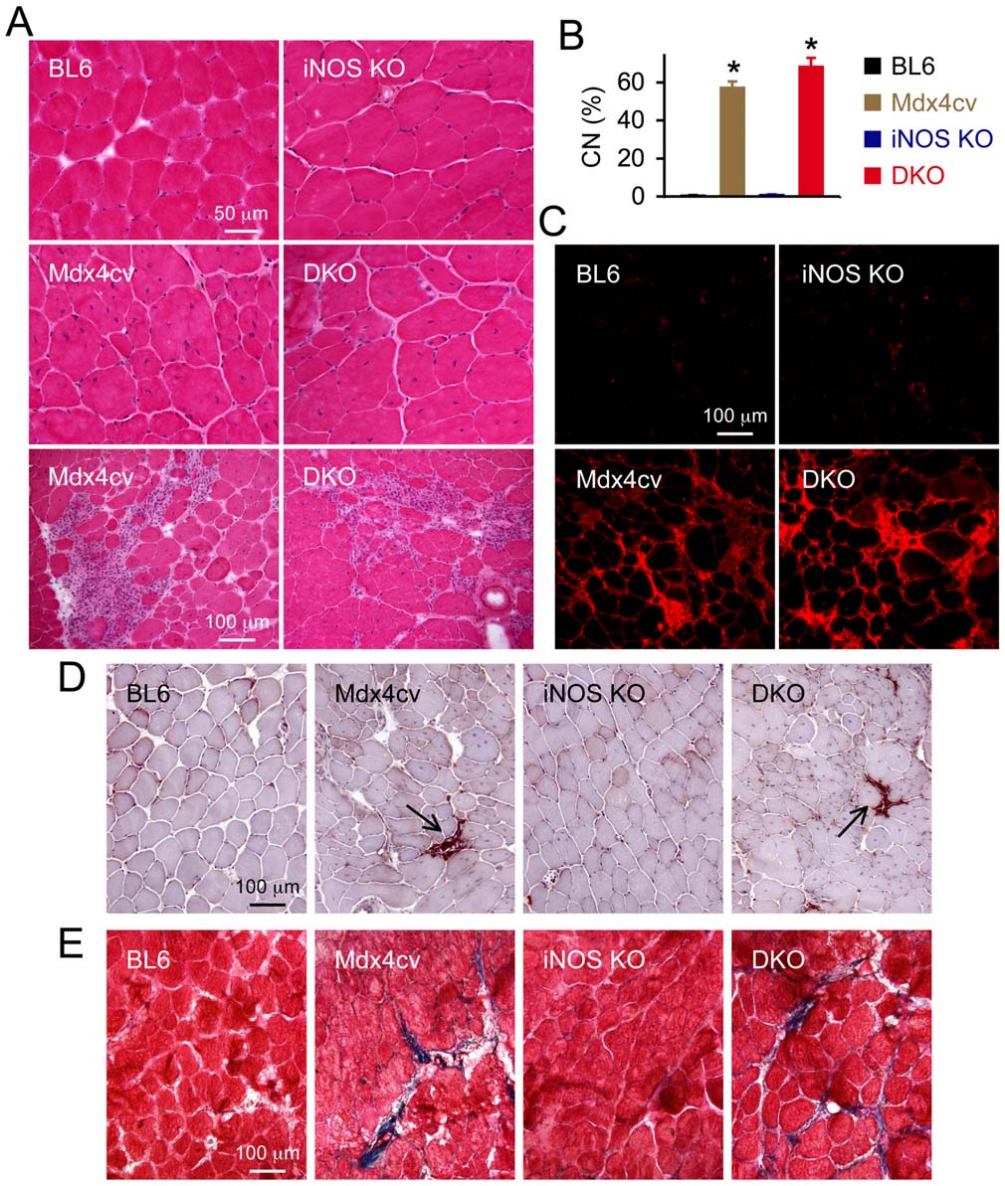
Genetic elimination of iNOS does not reduce limb muscle histopathology in adult mdx4cv mice. **A**, Representative HE staining photomicrographs. DKO, iNOS/dystrophin double knockout. Scale bar in BL6 image applies to top and middle panels. Middle panel, representative high power photomicrographs revealing central nucleation in mdx4cv and DKO mice; Bottom panel, representative low power photomicrographs showing muscle inflammation in mdx4cv and DKO mice. **B**, Quantification of myofiber with centrally localized nucleus. Asterisk, significantly higher than those of BL6 and iNOS knockout mice but there is no significant difference between mdx4cv and iNOS/Dys DKO mice neither is there a significant difference between BL6 and iNOS knockout mice. **C**, Representative mouse IgG immunostaining photomicrographs. Scale bar applies to all images. There is minimal IgG infiltration in BL6 and iNOS knockout. **D**, Representative histochemical staining of macrophages. Scale bar applies to all images. Arrow, dark brown stained macrophages. **E**, Representative Masson trichrome staining. Stripes of blue stained fibrotic tissues are evident in mdx4cv and iNOS/dystrophin double knockout mice.

HE staining was performed to evaluate overall histopathology changes (Figure 2A). BL6 and iNOS null mouse muscles showed uniform myofiber size and peripherally localized myonuclei (Figure 2A top panel). As expected, mdx4cv muscle displayed characteristic dystrophic pathology including variable myofiber size, profound central nucleation and patches of muscle inflammation (Figure 2A middle and bottom panels). Exactly the same histological lesions were seen in iNOS/Dys DKO mouse muscle (Figure 2A middle and bottom panels).

To study sarcolemmal integrity, we performed an in vivo IgG infiltration assay [14], [19], [27]. While minimal IgG infiltration was seen in BL6 and iNOS KO muscles, we observed profound IgG accumulation in mdx4cv and iNOS/Dys DKO muscles (Figure 2C). We also examined macrophage infiltration by immunohistochemical staining (Figure 2D) and non-specific esterase staining (data not shown). Similar levels of macrophage infiltration were found in mdx4cv and iNOS/Dys DKO muscles. To evaluate fibrosis, we performed Masson trichrome staining. Muscle fibrosis was not seen in BL6 and iNOS KO mice. In both mdx4cv and iNOS/Dys DKO, we observed stripes of blue stained fibrotic tissues (Figure 2E).

### iNOS knockout did not alter specific forces of the mdx4cv EDL muscle but resulted in a moderate protection against eccentric contraction-induced force decline

**Figure 3:**
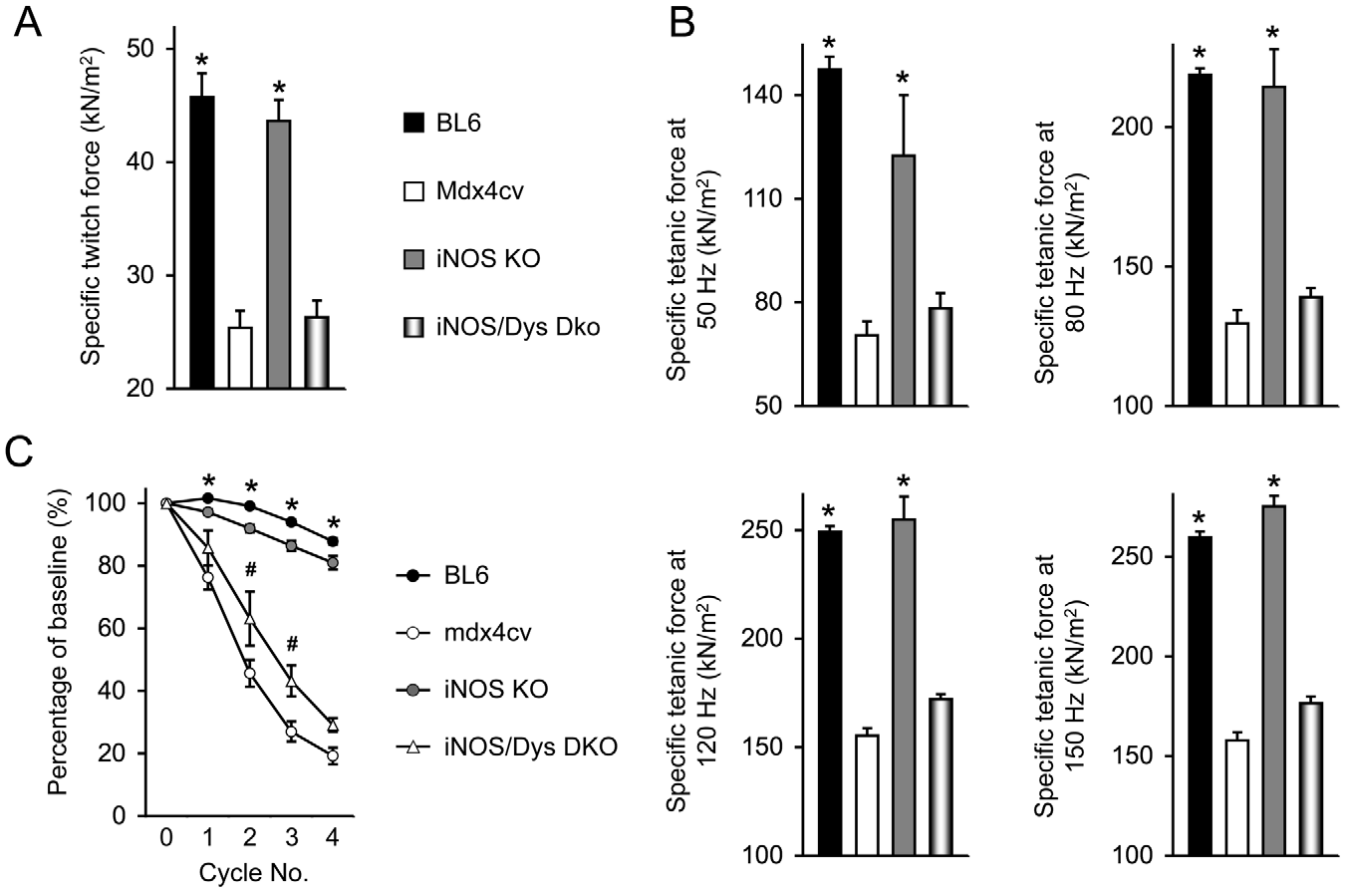
Characterization of EDL muscle contractility in iNOS/dystrophin double knockout mice. **A**, Specific twitch force. **B**, Specific tetanic forces at 50, 80, 120 and 150 Hz. **C**, Relative force decline following four cycles of eccentric contraction. Sample size, N = 8 for BL10, N = 11 for mdx4cv, N = 5 for iNOS KO, N = 4 for iNOS/Dys DKO. Asterisk, significantly higher than those of mdx4cv and iNOS/Dys DKO mice. However, there is no significant difference between BL6 and iNOS KO mice neither is there a significant difference between mdx4cv and iNOS/Dys DKO mice. Pound sign, value in iNOS/dystrophin double knockout mice is significantly higher than that of mdx4cv mice at the same round of eccentric contraction.

To study the physiological consequences of iNOS ablation on dystrophin-deficient muscle, we measured the specific twitch (1 Hz) force and specific tetanic forces under low (50 Hz), moderate (80 Hz) and high (120 and 150 Hz) stimulation frequencies (Figure 2A and 2B). No significant difference was observed between iNOS KO and BL6 mice. In mdx4cv and iNOS/Dys DKO mice, specific twitch and tetanic forces were significantly reduced. They only reached approximately 50 to 60% of those of normal mice (Figure 3A and B). Although iNOS/Dys DKO mice showed slightly higher numerical values of the specific tetanic forces than those of mdx4cv mice, the difference did not reach statistical significance (Figure 3B).

Next, we examined the force decline profile following repeated cycles of eccentric contraction. iNOS KO and BL6 mice showed similar profiles. In both cases, muscle force was largely preserved over four rounds of eccentric contraction (Figure 3C). Eccentric contraction resulted in significant force loss in both mdx4cv and iNOS/Dys DKO mice. However, the withholding forces of iNOS/Dys DKO mice were constantly higher than those of mdx4cv mice. Statistic significance was reached following two and three rounds of eccentric contraction (Figure 3C).

### iNOS ablation did not increase nNOS expression

**Figure 4:**
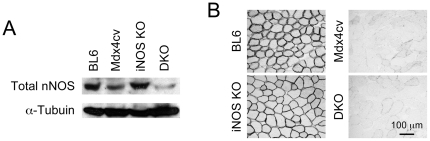
iNOS elimination does not augment nNOS expression. **A**, Representative western blot of total muscle nNOS. **B**, Representative photomicrographs of nNOS activity staining. Scale bar applies to all images. DKO, iNOS/dystrophin double knockout.

To determine whether iNOS knockout influences nNOS expression, we performed western blot, nNOS activity staining and nNOS immunofluorescence staining (Figure 4 and data not shown). Consistent with previous reports [33], we did not see a substantial elevation of the total nNOS level in iNOS KO and iNOS/Dys DKO muscle (Figure 4A). Sarcolemmal nNOS expression pattern was not altered either (Figure 4B).

### Evaluation of nitrosative stress markers

**Figure 5:**
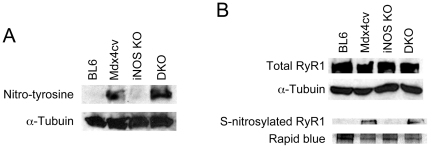
Characterization of nitrosative stress. **A**, Representative western blot analysis of 3-nitrotyrosine in BL6, mdx4cv, iNOS knockout and iNOS/dystrophin double knockout mice. α-Tubulin was used as the loading control. **B**, Representative western blots for total RyR1 (top panel) and S-nitrosylated RyR1 (bottom panel) in BL6, mdx4cv, iNOS knockout and iNOS/dystrophin double knockout mice. α-Tubulin was used as the loading control for total RyR1. Rapid blue staining was used as the loading control for S-nitrosylated RyR1.

We recently demonstrated that nNOS ablation reduced nitrosative stress in dystrophin-deficient muscle [14]. To determine whether iNOS knockout resulted in similar benefits, we examined nitrotyrosine, total RyR1 and S-nitrosylated RyR1 (Figure 5). In normal mice, neither nNOS knockout nor iNOS knockout changed nitrosative stress markers in muscle (Figure 5) [14]. While nitrosative stress markers were greatly diminished in nNOS/dystrophin double mutant mice [14], minimal differences were noted between mdx4cv and iNOS/Dys DKO mice (Figure 5).

## Discussion

Nitrosative stress-mediated RyR S-nitrosylation contributes to force reduction in DMD [6], [14]. Reactive nitrogen species derives from nitric oxide (NO), a short-lived, highly reactive molecule [34]. NO is synthesized by NOS. There are several types of NOS including nNOS, iNOS and endothelial NOS. nNOS is the predominant form in normal skeletal muscle [35]. It is anchored at the sarcolemma by collaborative action of dystrophin spectrin-like repeats 16/17 and syntrophin [15]. In the absence of dystrophin, nNOS delocalizes from the sarcolemma to the cytosol and the relative cytosolic NOS activity is substantially increased [14], [36], [37], [38]. As a consequence of pronounced muscle inflammation, iNOS expression is greatly increased in dystrophic muscle (Figure 1C and 2A, D) [6], [9], [10], [11]. To determine which NOS isoform is responsible for pathologic RyR S-nitrosylation and force inhibition, we created two different strains of double knockout mice (Figure 1) [14]. Both strains are based on dystrophin-null mdx4cv mice. Besides dystrophin deficiency, one strain carries a null mutation in the nNOS gene and the other strain carries a null mutation in the iNOS gene.

In nNOS/dystrophin double null (n-dko) mice, mislocalized cytosolic nNOS is completely removed [14]. At the same time, markers of nitrosative stress (such as nitro-tyrosine and RyR S-nitrosylation) were normalized in n-dko mice. Importantly, specific muscle forces were significantly enhanced [14].

Recently, Villalta et al generated iNOS/dystrophin-double null mice by crossing BL6 background iNOS KO mice with C57Bl/10 background mdx mice [9]. Interestingly, the authors focused their analysis on the soleus muscle, a muscle dominated by slow twitch myofibers. They observed reduced myofiber injury and reduced central nucleation but macrophage density and neutrophil number were not altered in the soleus muscle of iNOS-null mdx mice [9].

To exclude the confounding factor of the genetic background, we generated iNOS/Dys DKO mice in the same genetic background (BL6) (Figure 1). Since DMD preferentially affects fast twitch muscles (such as the EDL and TA muscles) [39], [40] and also since our previous studies were performed on the EDL muscle, here we opted to focus our study on the fast twitch muscle. We examined histopathology and also measured specific forces of the EDL muscle (Figures 2 and 3). Based on the findings of Villalta et al [9], we initially thought we should detect substantial reduction of muscle disease in iNOS/Dys DKO mice. Similar to Villalta et al, we did not see a dramatic change in macrophage infiltration (Figure 2D) [9]. However, analysis of multiple aspects of histological lesions (central nucleation, sarcolemmal integrity and muscle fibrosis) did not yield convincing evidence of muscle disease amelioration (Figure 2). According to the model of iNOS-mediated RyR S-nitrosylation [6], we initially expected iNOS/Dys DKO mice to produce significantly higher specific force than mdx4cv mice. Surprisingly, genetic elimination of the iNOS gene did not alter contractility of the EDL muscle in mdx4cv mice (Figure 3). The slight difference in the numerical values of specific tetanic forces was apparently due to the difference in the muscle weight and CSA (Figure 3, Table 1). Additional studies suggest that iNOS ablation did not alter nNOS expression neither did it reduced nitrosative stress markers in iNOS/Dys DKO mice (Figures 4 and 5).

The results from our n-dko mice and iNOS/Dys DKO mice suggest that mislocalized nNOS, rather than elevated iNOS, may play a determining role in nitrosative modification of RyR and force decline in dystrophin-deficient muscle (Figures 3 and 5) [14]. In contrast to nNOS, iNOS activation is not dependent on calcium [7], [41], [42]. This also seems to fit our model. Considering the fact that resting intracellular calcium concentration is abnormally elevated in DMD muscle [43], it is perceivable that there may exist a positive feedback loop between S-nitrosylated leaky RyR channel and cytosolic nNOS activation. On the other side, the moderate improvement of the eccentric contraction profile of iNOS/Dys DKO mice suggests that elevated iNOS remains a detrimental insult in DMD [9].
